# The effects of daptomycin on cell wall biosynthesis in *Enterococcal faecalis*

**DOI:** 10.1038/s41598-023-39486-8

**Published:** 2023-07-28

**Authors:** Binayak Rimal, James Chang, Chengyin Liu, Raiyan Rashid, Manmilan Singh, Sung Joon Kim

**Affiliations:** 1 Institute of Biomedical Studies, Baylor University, Waco, TX 76798 USA; 2 Department of Chemistry One Bear Place #97046, Baylor University, Waco, TX 76798 USA; 3 Department of Chemistry, Howard University, Washington, DC 20059 USA; 4 Department of Chemistry, Washington University, St. Louis, MO 63110 USA; 5 Division of Infectious Diseases, Johns Hopkins University School of Medicine, Baltimore, MD 21287 USA

**Keywords:** Antibiotics, Solution-state NMR

## Abstract

Daptomycin is a cyclic lipodepsipeptide antibiotic reserved for the treatment of serious infections by multidrug-resistant Gram-positive pathogens. Its mode of action is considered to be multifaceted, encompassing the targeting and depolarization of bacterial cell membranes, alongside the inhibition of cell wall biosynthesis. To characterize the daptomycin mode of action, ^15^N cross-polarization at magic-angle spinning NMR measurements were performed on intact whole cells of *Staphylococcus aureus* grown in the presence of a sub-inhibitory concentration of daptomycin in a chemically defined media containing l-[ϵ-^15^N]Lys. Daptomycin-treated cells showed a reduction in the lysyl-ε-amide intensity that was consistent with cell wall thinning. However, the reduced lysyl-ε-amine intensity at 10 ppm indicated that the daptomycin-treated cells did not accumulate in Park’s nucleotide, the cytoplasmic peptidoglycan (PG) precursor. Consequently, daptomycin did not inhibit the transglycosylation step of PG biosynthesis. To further elucidate the daptomycin mode of action, the PG composition of daptomycin-susceptible *Enterococcus faecalis* grown in the presence of daptomycin was analyzed using liquid chromatography-mass spectrometry. Sixty-nine muropeptide ions correspond to PG with varying degrees of modifications including crosslinking, acetylation, alanylation, and 1,6-anhydrous ring formation at MurNAc were quantified. Analysis showed that the cell walls of daptomycin-treated *E. faecalis* had a significant reduction in PG crosslinking which was accompanied by an increase in lytic transglycosylase activities and a decrease in PG-stem modifications by the carboxypeptidases. The changes in PG composition suggest that daptomycin inhibits cell wall biosynthesis by impeding the incorporation of nascent PG into the cell walls by transpeptidases and maturation by carboxypeptidases. As a result, the newly formed cell walls become highly susceptible to degradation by the autolysins, resulting in thinning of the cell wall.

## Introduction

**Figure 1:**
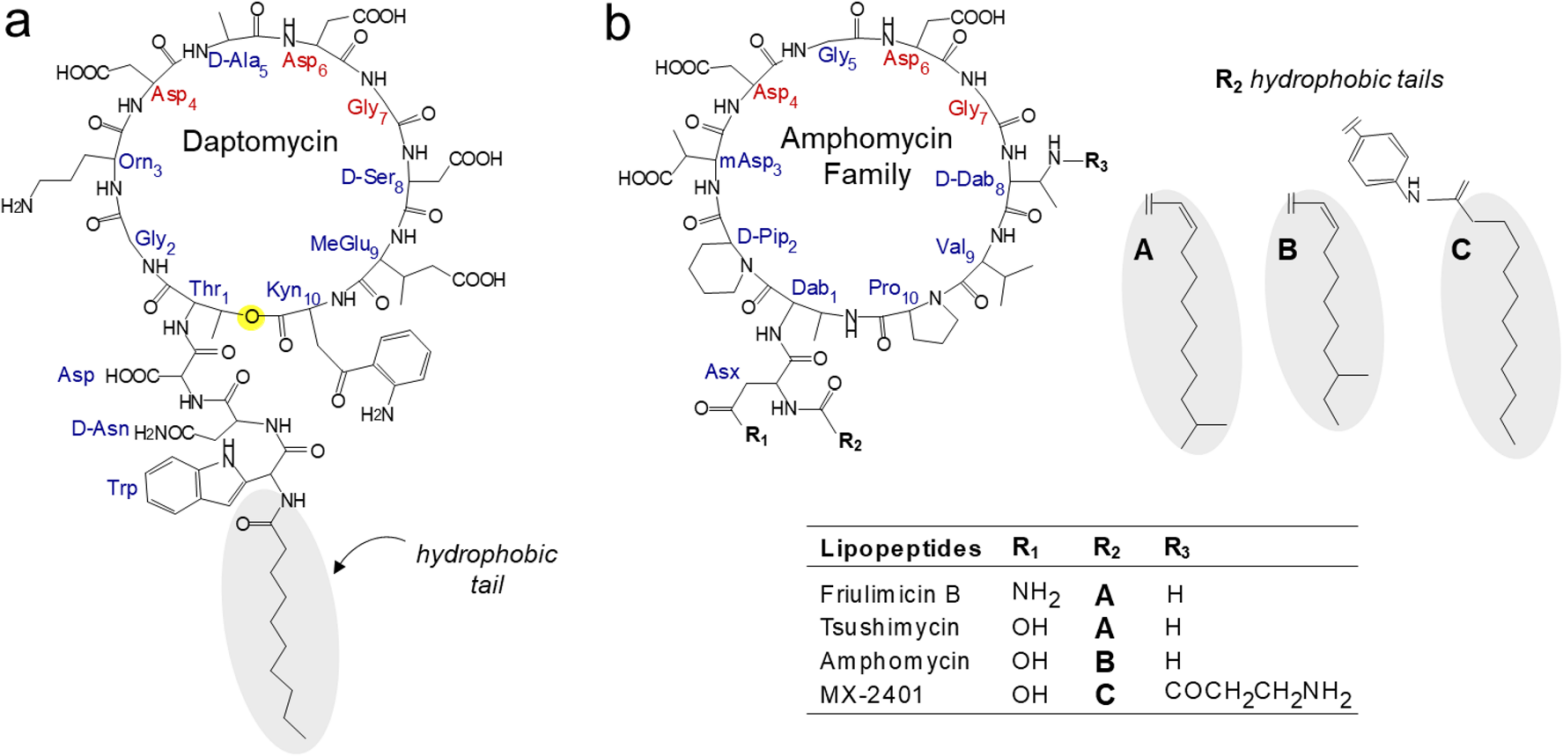
Chemical structure of decapeptide cyclic antimicrobial peptides: (**a**) daptomycin, (**b**) friulimicin B, amphomycin, and MX-2401. All cyclic decapeptides have a 10-amino acid cyclic-core structure with a conserved Asp_4_-X_5_-Asp_6_-Gly_7_ motif (highlighted in red), where X_5_ can be either a Gly or d-Ala. All cyclic antimicrobial peptides have a hydrophobic sidechain (highlighted gray oval) attached to the peptide-core structure.

Daptomycin, formally designated as LY146032 by Eli Lilly, is a cyclic lipopeptide^1^ discovered in the early 1980s from the fermentation broth of *Streptomyces roseosporus*. Daptomycin possesses a broad-spectrum antibacterial activity against Gram-positive pathogens^2^. With the emergence of multidrug resistance, daptomycin became the first in a new class of antibiotics that was approved by the FDA in 2003 for the treatment of serious infections^3–5^. The chemical structure of daptomycin (Fig. 1a) shares numerous structural similarities with the amphomycin family of lipopeptide antibiotics, which includes amphomycin, friulimicin, MX-2401, tsushimycin, and A54145 (Fig. 1b). The shared structural similarities include a cyclic-core structure consisting of ten amino acids to which a hydrophobic sidechain is attached (Fig. 1, gray oval). Both daptomycin and amphomycin antibiotics exhibit calcium-dependent activities and possess a conserved Asp_4_-X_5_-Asp_6_-Gly_7_ motif that functions as a calcium-ion binding site^6^. In daptomycin, the amino acid at position X_5_ is d-Ala, whereas in amphomycin it is a Gly. However, a key structural difference between daptomycin and amphomycin is that daptomycin is a cyclic depsipeptide with an ester bond between the amino acids Thr_1_ and Kyn_10_ (Fig. 1a, yellow circle), whereas, in amphomycin, a peptide bond connects the amino acids Dab_1_ and Pro_10_.

Despite structural similarities between amphomycin and daptomycin, their mode of action differs significantly. In the case of amphomycin (Fig. 1b), the addition of antibiotics to *S. aureus* during the mid-exponential growth resulted in cell wall thinning and the accumulation of Park’s nucleotide^7^. This effect is reminiscent of vancomycin^8^, a well-known transglycosylase inhibitor that disrupts peptidoglycan (PG) biosynthesis by binding to lipid II, a membrane-bound PG precursor^9–11^. In contrast, daptomycin primarily targets bacterial membranes. In vitro assays have shown that daptomycin readily bind to lipid vesicles in a calcium-dependent manner^12^. The daptomycin-Ca^2+^ complex exhibits high binding affinity to the negatively charged phosphoglycerol headgroup of lipids present in the membrane^13^. The membrane-bound daptomycin is thought to form an aggregate^12^ and insert its hydrophobic sidechain (Fig. 1, gray oval) into the lipid bilayer to form a pore structure that leads to potassium ion leakage^14^. This membrane depolarization has been attributed to daptomycin’s potent bactericidal activity. However, the bacterial membrane depolarization has been shown to occur only at high concentrations of daptomycin. In the case of *Staphylococcus simulans*, the concentration of daptomycin required to induce membrane depolarization exceeded the minimum inhibitory concentration (MIC) by more than tenfold^11^. Furthermore, even at such elevated daptomycin concentrations, membrane depolarization only ensued after a five-minute incubation period. Similar high daptomycin concentration requirements for the membrane disruption of *S. aureus* and *Bacillus subtilis*^15^ suggest that the daptomycin mode of action at low concentrations, such as at MIC, is likely to be complex without necessarily involve the formation of membrane pores.

Daptomycin, when present at low concentrations, seems to interfere with cell wall biosynthesis as the addition of daptomycin to *B. subtilis* results in cell growth with aberrant cell wall morphology and defective septum formation^16^. Furthermore, when the fluorescence-labeled daptomycin is introduced to growing *S. aureus*, it does not distribute uniformly throughout the cell but instead it localizes specifically to the divisional septum, which is the site of PG biosynthesis. This localized binding suggests that daptomycin’s interaction with the membrane can disrupt or mislocalization of cell division proteins, impacting both cell division and cell wall biosynthesis. An alternative hypothesis is that daptomycin can directly bind to lipid II^15^, a membrane-bound PG precursor, forming a tripartite daptomycin-Ca^2+^-lipid II complex^17^. This hypothesis is supported by observations that daptomycin’s binding to the lipid bilayer increases significantly in the presence of lipid II. Additionally, the preincubation of daptomycin with lipid II prevented the lipodepsipeptide from binding to *S. aureus* to result in delayed killing.

In this study, the mode of action of daptomycin was investigated using solid-state NMR and liquid chromatography-mass spectrometry (LC–MS). Solid-state NMR was used to examine the proposed inhibition of the transglycosylation step of PG biosynthesis by daptomycin. This was achieved by monitoring the changes in Park’s Nucleotide accumulation in intact whole cells of *S. aureus* grown in the presence of daptomycin at sub-inhibitory concentrations. This is a well-established method where *S. aureus* has been routinely used for elucidating the mode of action of transglycosylase inhibitor targeting lipid II, including vancomycin^18–21^. If daptomycin binds to lipid II, it would prevent the regeneration of the lipid transporter from lipid II by the transglycosylase, resulting in a large accumulation of Park’s Nucleotide in the cytoplasm of *S. aureus*.

In order to gain further insight into the impact of daptomycin on cell wall biosynthesis, the changes in the PG composition of *Enterococcus faecalis* grown in the presence or absence of daptomycin were analyzed using LC–MS. *E. faecalis* was chosen due to its clinical significance, as daptomycin is commonly used for treating *E. faecalis* infections that are unresponsive to vancomycin therapy. Additionally, daptomycin resistance in *E. faecalis* has been linked to two enzymes that are involved in phospholipid metabolism and three-component regulatory systems known as LiaFSR, which regulates cell envelope stress response^22^. In daptomycin-resistant *E. faecalis*, these regulatory systems affect the lipid composition of the membrane^23^ and it is highly likely that they also affect the PG composition of the cell walls. Hence, comprehensive PG composition analysis using LC–MS is crucial for understanding the mode of action of daptomycin and provides valuable insight into the underlying mechanisms of resistance in *E. faecalis*.

## Results and discussion

### Addition of daptomycin to static cells

The impact of daptomycin on the membrane permeability was assessed using an ATP-leakage assay. Varying concentrations of daptomycin were added to overnight cultures of *S. aureus* and *E. faecalis* that were resuspended in phosphate-buffered saline (PBS) supplemented with 20 mM Ca^2^. After a 20-min incubation at 37 °C, ATP leakage was evaluated by introducing 100 μL of CellTiter-Glo 2.0 reagent containing luciferin and luciferase, which catalyzes ATP-dependent oxidative decarboxylation of luciferin, resulting in the releases photons. The extent of ATP leakage was determined by measuring the luminescence intensity at 560 nm for 200 ms.

**Figure 2:**
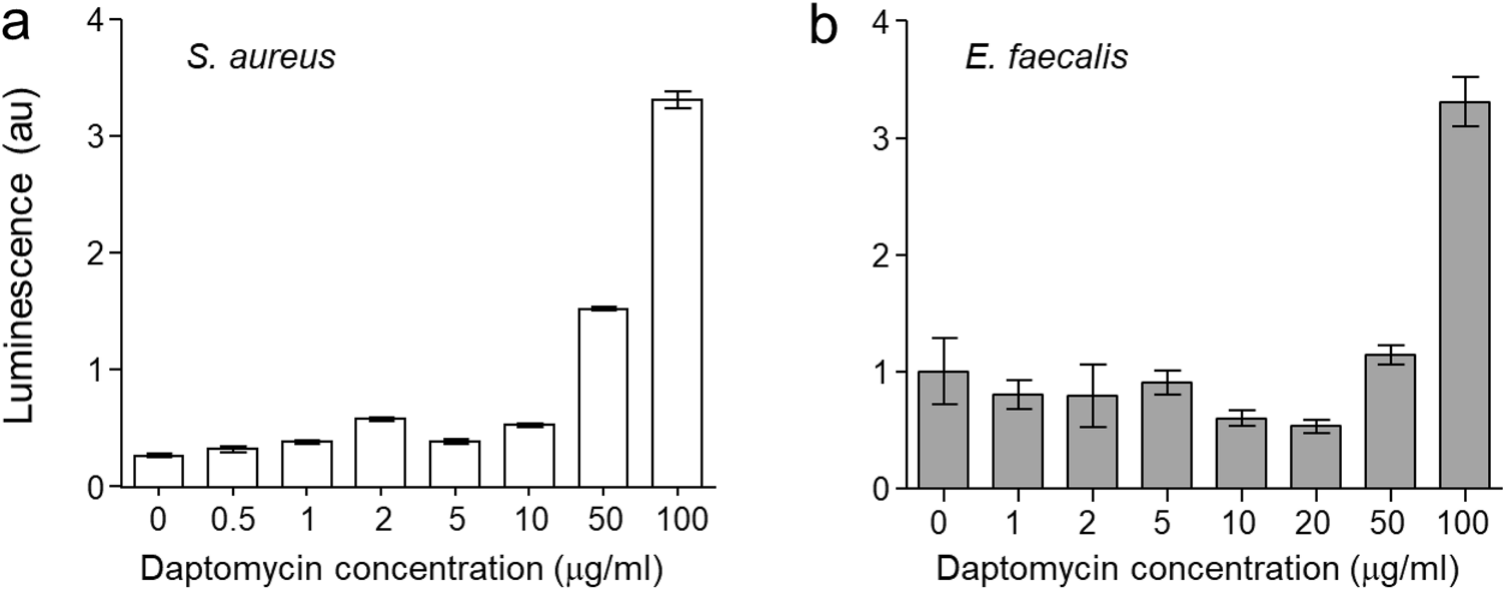
Daptomycin-induced ATP leakage in nondividing cells of *S. aure*us and *E. faecalis*. (**a**) ATP leakage assay of static *S. aureus*^*9*^ is reproduced from reference 7. (**b**) Nondividing *E. faecalis* was prepared by harvesting cells at OD_600_ of 1.5 and resuspended in PBS supplemented with 20 mM Ca^2+^. Daptomycin was added to a final concentration of 0, 1, 2, 5, 10, 20, 50, and 100 µg/mL and incubated at 37 °C for 20 min. Following the addition of 100 μL of CellTiter-Glo 2.0 reagent, ATP leakage was detected and quantified by integrating the intensity of luminescence at 560 nm for 200 ms. Daptomycin-induced membrane disruption was observed only at the highest concentration of 100 µg/mL for both *S. aureus* and *E. faecalis*. Error bars represent a 95% confidence interval (n = 8).

A previous study has shown that daptomycin exhibits potent bactericidal activity against the nondividing cells of *S. aureus* through membrane depolarization^24^. However, we observed that daptomycin did not exhibit significant ATP leakages again nondividing cells of both *S. aureus*^9^ and *E. faecalis*, except at a high concentration of 100 μg/mL (Fig. 2). Although the absence of ATP leakage does not necessarily imply the absence of pore formation, transmission electron microscopy (TEM) images of *S. aureus* treated with daptomycin at a low concentration did not reveal any sign of cell lysis or abnormal cell growth. These findings suggest that the mode of action of daptomycin at low concentrations differs from that at high concentrations and does not involve the formation of membrane pores^25^.

### Effects of daptomycin on *S. aureus* cell wall biosynthesis characterized using solid-state NMR

**Figure 3:**
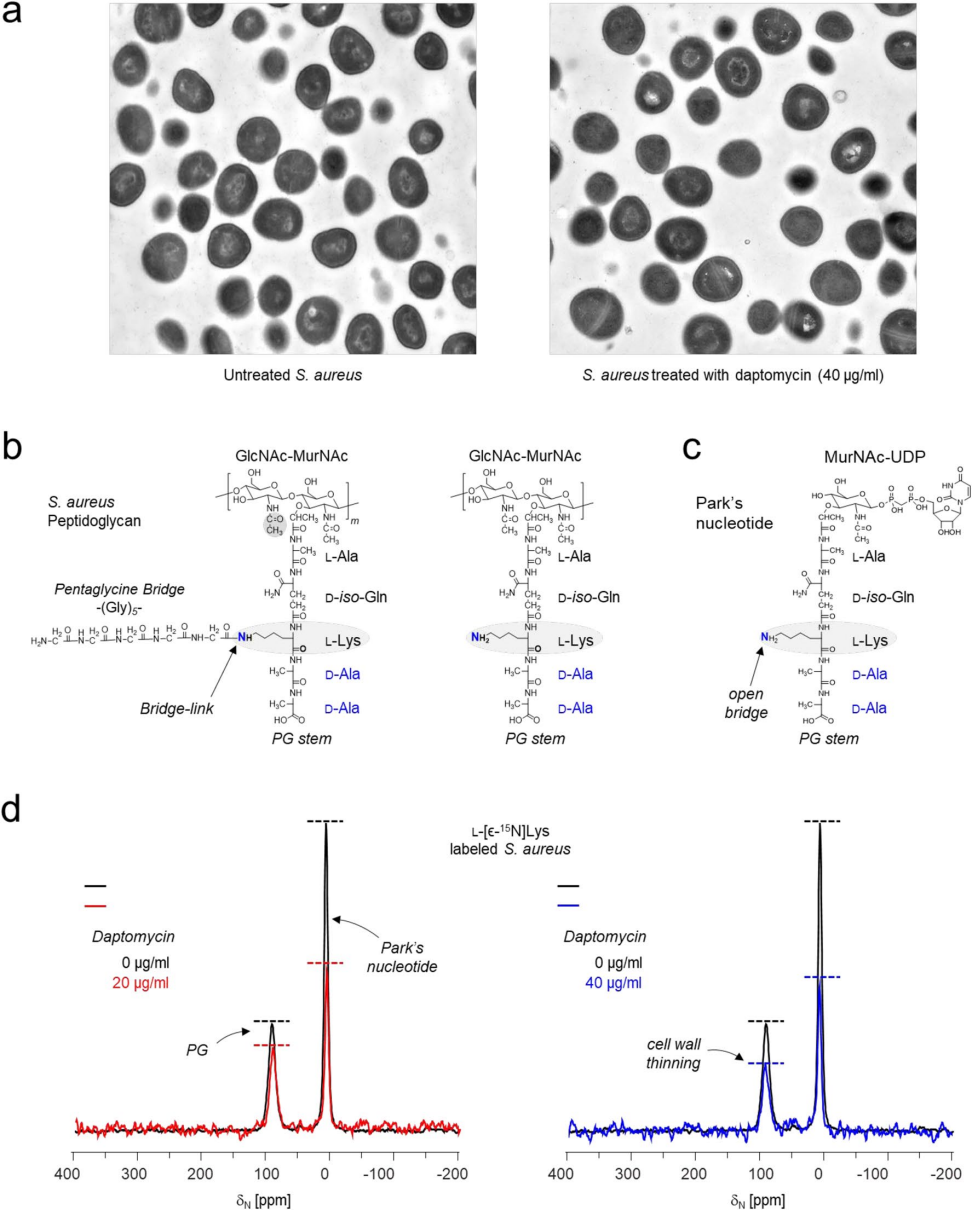
Daptomycin addition to growing *S. aureus*. (**a**) Transmission electron micrograph images of *S. aureus* that were untreated (left) and treated with antibiotic by the addition of daptomycin to a final concentration of 40 µg/mL during the mid-exponential growth phase (right). (**b**) Chemical structure of peptidoglycan in *S. aureus*. The isotope-labeled l-[ϵ-^15^N]Lys incorporated in the PG is highlighted in grey with the ^15^N isotope shown in blue. (**c**) Chemical structure of Park’s Nucleotide, a cytoplasmic PG precursor. (**d**) ^15^N-CPMAS spectra of intact whole cells of *S. aureus* (ATCC 6538P) grown in chemically defined media containing l-[ϵ-^15^N]Lys. Left) ^15^N-CPMAS spectra of *S. aureus* after daptomycin was added during the mid-exponential growth to a final concentration of 0 (black line) and 20 μg/mL (red line). Right) ^15^N-CPMAS spectra of *S. aureus* treated with daptomycin to final concentrations of 0 (black line) and 40 μg/mL (blue line). Each spectrum is a result of 20 k scans. The spectra are normalized to equal sample weight and the number of scans. Magic angle spinning was 5 kHz.

To investigate the mode of action of daptomycin at sub-inhibitory concentrations, three samples of intact whole cells of *S. aureus* (ATCC 6538P) were prepared and analyzed using solid-state NMR. Samples were prepared by grown *S. aureus* in a chemically defined media^26–28^ containing stable-isotope labeled amino acids [^13^C]Gly and l-[ϵ-^15^N]Lys. Daptomycin was introduced during the mid-exponential growth at an optical density of 0.4 at 600 nm (OD_600_ 0.4), together with a Ca^2+^ concentration of 50 μg/mL, reaching final concentrations of 0, 20, or 40 μg/mL. After 90 min of additional growth, the cells were harvested and lyophilized for the analysis. A small aliquot (5 ml) of the culture was retained to monitor ongoing growth, which showed that the addition of daptomycin at 20 and 40 μg/mL did not inhibit the growth of *S. aureus*. The TEM images of *S. aureus* treated with daptomycin at 40 μg/mL showed no noticeable signs of aberrant cell morphology or membrane disruption (Fig. 3a).

During mid-exponential growth, the provisioned l-[ϵ-^15^N]Lys is actively taken up by the bacteria and incorporates into the PG-stem structure of *S. aureus*^28^. The chemical structure of the PG-repeat unit (Fig. 3b, left) consists of the disaccharide GlcNAc-MurNAc with a pentapeptide stem l-Ala–d-iso-Gln–l-Lys–d-Ala–d-Ala, and a pentaglycine bridge attached to the ϵ-nitrogen on the l-Lys sidechain of the stem structure. Approximately 85% of the PG stems in *S. aureus* cell walls are found with an attached bridge unit, and the remaining 15% with an open bridge (Fig. 3b, right)^26^. The ^15^N-labeled l-[ϵ-^15^N]Lys is also actively incorporated into Park’s nucleotide (Fig. 3c) which is the cytoplasmic PG precursor with an open bridge structure (Fig. 3c).

The addition of daptomycin at sub-inhibitory concentration inhibited the PG biosynthesis in *S. aureus*. This effect is clearly visible in Fig. 3d which shows the ^15^N cross-polarization at magic angle spinning (CPMAS) NMR spectra of *S. aureus* treated with daptomycin at 20 μg/mL (red line) and at 40 μg/mL (Fig. 3d, blue line). The spectra are overlaid with the spectrum of untreated cells (black). In the spectra, the peak at ^15^N-chemical shift of 95 ppm corresponds to the lysyl-ε-amide of l-[ϵ-^15^N]Lys found in PG units with a pentaglycine bridge unit attached^26,29^. A peak at 10 ppm corresponds to lysyl-ε-amine found in proteins, PG with an open bridge link, and Park’s nucleotide^26^. The overlaid spectra clearly exhibits a concentration-dependent reduction in the intensities of lysyl-ε-amide at 95 ppm. This decrease indicates a significant reduction in the number of PG units with an attached bridge, which is consistent with cell wall thinning in daptomycin-treated *S. aureus*. Similar effects of cell wall thinning has been observed when *S. aureus* were treated with known antibiotics targeting cell wall biosyntheses, including vancomycin^26,29^, amphomycin, plusbacin A_3_^30^, and oritavancin^18,31^. Furthermore, the decreased intensity of lysyl-ε-amine resonance at 10 ppm shows that the cytoplasm of daptomycin-treated *S. aureus* does not accumulate in Park’s Nucleotide. Therefore, daptomycin does not target the transglycosylation step of PG biosynthesis.

### Addition of daptomycin to *E. faecalis* during growth

**Figure 4:**
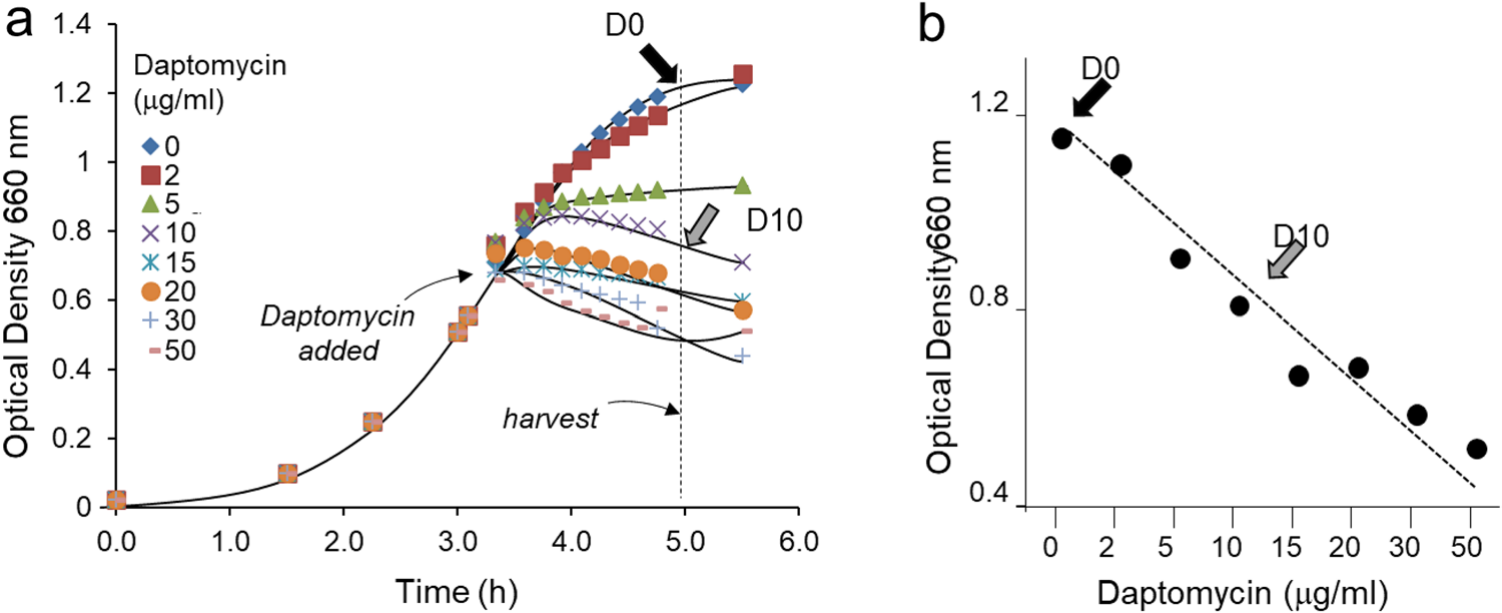
Addition of daptomycin to *E. faecalis* during growth. (**a**) Growth inhibition of *E. faecalis* by daptomycin. Daptomycin was added during the mid-exponential growth phase at an OD_600_ of 0.55 and the bacteria were harvested after 90 min of growth in presence of the antibiotic. For the liquid chromatography-mass spectrometry (LC–MS) analysis, cell walls were isolated from *E. faecalis* grown in the absence of daptomycin (black arrow, D0) and in the presence of daptomycin at 10 µg/mL (gray arrow, D10). (**b**) The growth inhibition of *E. faecalis* by daptomycin was assessed by measuring OD_600_ after 5 h growth, and the plot shows daptomycin exhibits concentration-dependent inhibition of *E. faecalis*.

The growth inhibitory effect of daptomycin on *E. faecalis* in brain heart infusion (BHI) media during the mid-exponential growth was monitored by measuring the OD_600_ (Fig. 4a). The addition of daptomycin at OD_600_ of 0.55 resulted in a concentration-dependent inhibition of growth (Fig. 4b). *E. faecalis* treated with daptomycin at 10 µg/mL, which showed strong growth inhibition, was selected for LC–MS analysis. Two samples of isolated cell walls of *E. faecalis* were prepared: 1) grown in the absence of antibiotic (Fig. 4, marked with a black arrow and labeled as D0) and 2) grown in the presence of daptomycin at 10 µg/mL (marked with a gray arrow and labeled as D10).

### Identification and quantification of *E. faecalis* muropeptide fragments by LCMS

**Figure 5:**
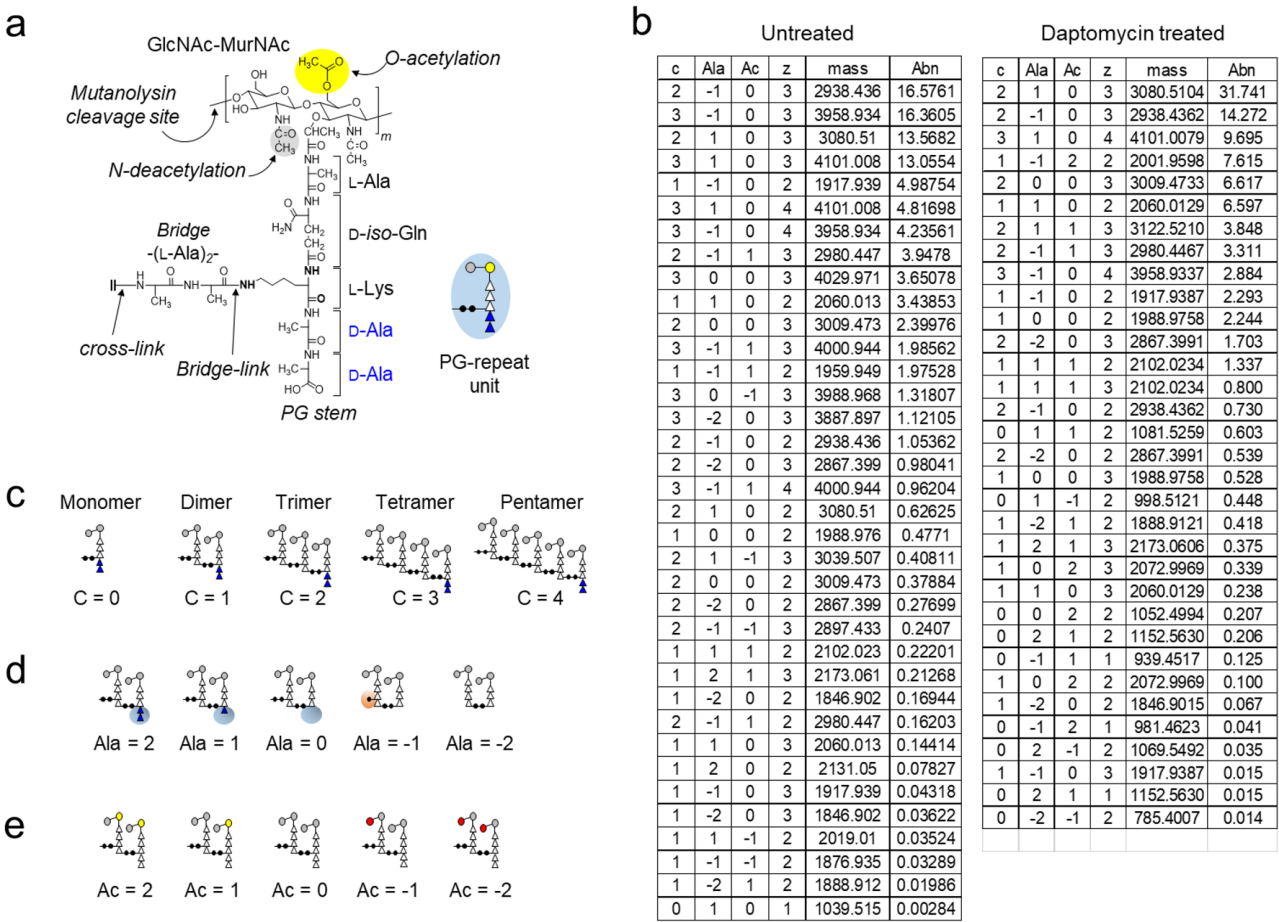
Structure of an *E. faecalis* peptidoglycan subunit and observed muropeptide fragments. (**a**) Chemical structure of a PG repeat unit in *E. faecalis* with a schematic figure representing a repeat unit shown as an inset. The disaccharide, GlcNAc-MurNAc, is represented by grey circles. A pentapeptide stem unit composed of l-Ala-d-iso-Gln-l-Lys-d-Ala-d-Ala (five triangles) is attached to the MurNAc of the disaccharide. The l-Ala-l-Ala bridge (filled circles) is attached to the side chain of l-Lys and forms a crosslink with carbonyl carbon of d-Ala at the fourth position from the neighboring repeat unit’s stem. Mutanolysin cleaves the GlcNAc-MurNAc β-1,4 glycosidic linkage in PG and generates the fragments. (**b**) Mutanolysin-digested muropeptide fragments characterized by LC–MS. Thirty-six muropeptide ions from the wild type (untreated) and thirty-three from daptomycin-treated cells were identified from LC–MS, and each muropeptide was quantified by integrating the extracted ion chromatogram (XIC) of the selected muropeptide ion. Every identified muropeptide ion is described by its crosslinking (**c**), the number of terminal d-Ala (Ala), acetylation state (Ac), charge state (z), and abundance (Abn). The muropeptides are listed in the order of descending abundance. (**c**–**e**) Muropeptide oligomers with an increasing number of crosslinks (0 to 4), alanine (− 2 to + 2), and acetylation state (− 2 to 2) are schematically depicted, respectively.

PG-repeat unit of *E. faecalis* differs structurally from *S. aureus* due to its crosslinking bridge. In *S. aureus* the bridge is a pentaglycine, whereas in *E. faecalis*, it is an l-Ala-l-Ala dipeptide. The crosslinking of PG units is formed through a peptide bond between the amine of the l-Ala bridge from the acceptor stem and the carbonyl carbon of the penultimate d-Ala in the donor stem of the neighboring glycan chain (Fig. 5a).

For the analysis, isolated cell walls of daptomycin-susceptible *E. faecalis* were prepared from cultures grown with and without daptomycin. The hydrofluoric acid treatment of isolated cell walls, which would remove wall-teichoic acid covalently attached to the PG, was omitted in order to preserve the O-acetylation of PG. Prior to LCMS analysis, the isolated cell walls were digested using mutanolysin, an enzyme that cleaves the β-1,4 glycosidic linkage between GlcNAc and MurNAc (Fig. 5a). This enzymatic digestion resulted in a complex mixture of muropeptide fragments with various PG modifications, which were then separated using liquid chromatography.

To identify the muropeptide fragments, high-resolution mass spectrometry was used to determine the accurate mass of muropeptides. The observed masses were then matched to the entries from the in silico muropeptide library that was generated using an in-house program^32^. A total of sixty-nine muropetide ion species were included in the analysis, with thirty-six from the untreated and thirty-three from the daptomycin-treated PG of *E. faecalis*. The relative amount of each muropeptide fragment was quantified by integrating the extracted ion chromatogram (XIC) of the selected muropeptide ion^33^. Isotopic distribution and necessary correction of abundance for each identified ion were calculated using a MATLAB program developed in-house. Figure 5b shows various muropeptide fragments observed by LC–MS analysis along with their quantified relative abundances. These muropeptide ions are categorized according to their crosslinking (c), the number of terminal d-Ala (Ala), acetylation state (Ac), charge state (z), and abundance (Abn).

### Effects of daptomycin on *E. faecalis* PG crosslinking

**Figure 6:**
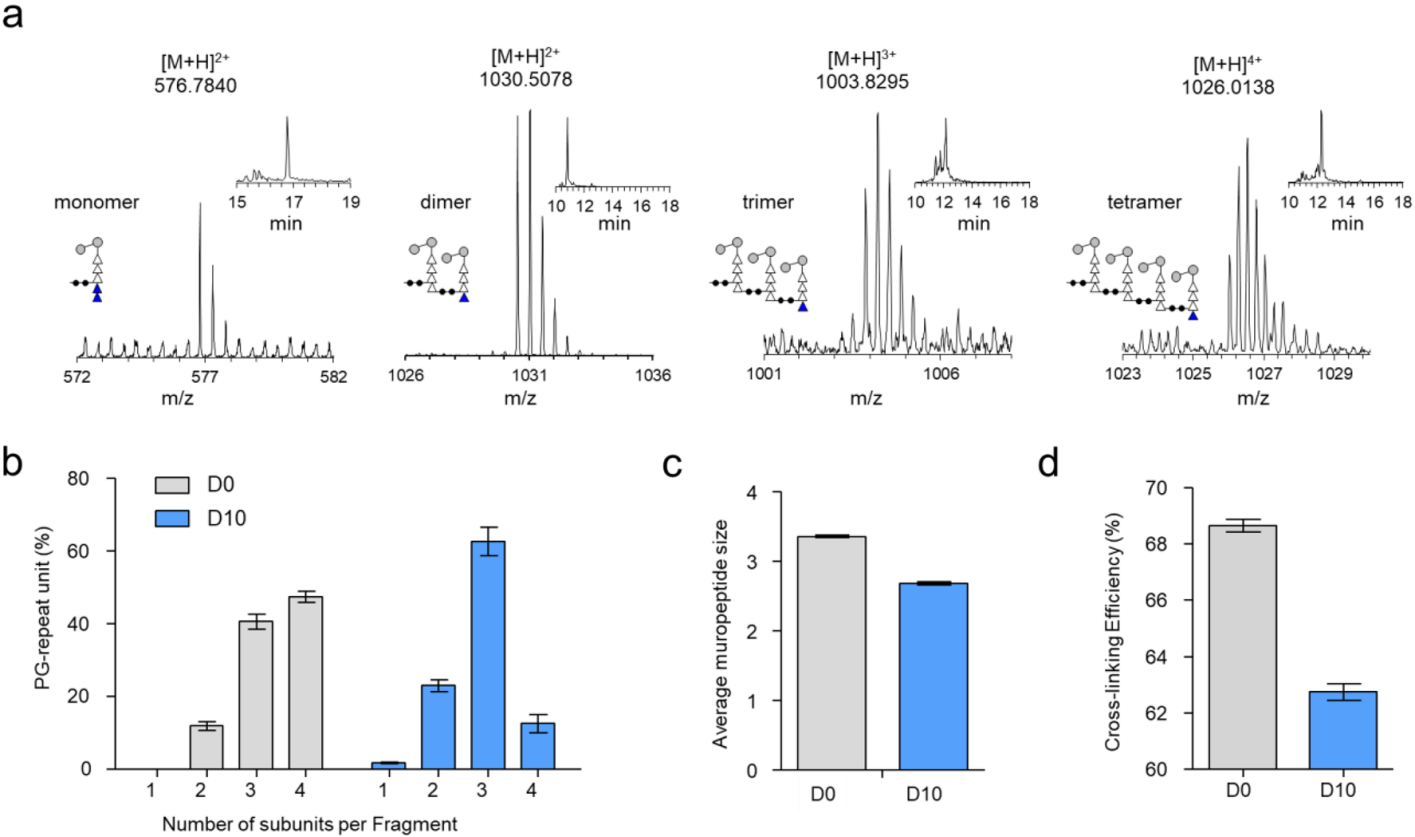
Daptomycin treatment of *E. faecalis* reduces PG crosslinking. (**a**) Mass spectra of PG monomers, dimers, trimers, and tetramers derived from mutanolysin-digested cell walls of daptomycin-treated *E. faecalis*. Corresponding extracted ion chromatograms and schematic representations of the muropeptide structure and XIC are shown as insets. (**b**) All identified muropeptide species were categorized based on the number of repeat disaccharide units. Quantitative analysis shows that PG tetramers constituted as the most abundant muropeptides in the untreated cell walls, accounting for 47.49% ± 1.13% (gray, D0). Upon the addition of daptomycin at 10 µg/mL (blue, D10), a decrease in tetramers and an increase in trimers were observed, indicating reduced PG crosslinking. The daptomycin-treated sample shows a high abundance of trimers at 62.71% ± 3.99%. The t-test showed significant differences between untreated and treated cell walls for monomers, dimers, trimers, and tetramers, with p-values of 4.0 × 10^–6^, 2.1 × 10^–5^, 2.9 × 10^–5^, and 1.0 × 10^–6^, respectively. Muropeptide fragments larger than tetramers were not observed. The percent PG-repeat unit composition is provided in Table S1a. (**c**) Calculated average muropeptide size for untreated *E. faecalis* was 3.36 ± 0.02 PG-repeat units, while daptomycin-treated *E. faecalis* was 2.69 ± 0.02 PG-repeat units (Table S1b). The calculated average muropeptide size is provided in Table S1b. (**d**) Calculated PG crosslinking efficiencies of *E. faecalis* grown in the absence and presence of daptomycin at 10 µg/mL (Table S1c). PG crosslinking decreases for OG1RF upon treatment with daptomycin (*p* < 0.001). All error bars represent a 95% confidence interval (n = 3).

The intensity of muropeptide ions, identified by m/z values, was analyzed based on oligomeric size to determine the degree of PG crosslinking (Fig. 5c). Figure 6a shows representative mass spectra of the PG monomer, dimer, trimer, and tetramer from mutanolysin-digested isolated cell walls of *E. faecalis* grown without daptomycin. The corresponding XIC and matching schematic representation of the muropeptide for each spectrum are shown as figure insets. The muropeptide distribution in the cell wall of *E. faecalis* grown without daptomycin (gray bars, D0) is primarily composed of tetramers and trimers, but without monomers. The muropeptide sizes 1, 2, 3, and 4 refer to monomers, dimers, trimers, and tetramers, respectively. Muropeptide fragments larger than tetramers were not observed. In the presence of daptomycin (10 µg/mL), the composition *E. faecalis* PG undergoes significant changes, characterized by a large decrease in tetramers (from 47 to 13%) accompanied by increases in monomer, dimers, and trimers (Fig. 6b). The reduced PG crosslinking in the cell walls of *E. faecalis* following the daptomycin treatment is reflected in the decrease of an average muropeptide size, calculated as the weighted sum of the muropeptide distribution (Fig. 6b). The calculated average muropeptide size for the untreated *E. faecalis* was 3.4, and for daptomycin-treated *E. faecalis* was 2.7 PG-repeat units (Fig. 6c). The decreased average muropeptide size indicates a reduction in PG crosslinking efficiency within the cell walls of *E. faecalis* due to daptomycin treatment.

PG crosslinking efficiency (ρ_CL_) is defined as a percent of all subunits with crosslinks present^34^. PG ρ_CL_ was calculated by summing the integrated ion current (XICs) of muropeptide species and then dividing it by the total number of crosslinks as previously described^32^. For isolated cell walls of *E. faecalis* grown in absence of daptomycin, the calculated ρ_CL_ by LC–MS was at 69% (Fig. 6d). This is value is higher than the ρ_CL_ of 48% measured for intact whole cells using solid-state NMR^35^, as whole cells contain both mature and immature cell walls. Therefore, the calculated ρ_CL_ of 69% by LC–MS indicates high level of PG crosslinking in the mature cell walls of *E. faecalis*. However, when *E. faecalis* is grown in the presence of daptomycin (10 µg/ml), the ρ_CL_ for reduces to 63%. This large reduction in PG crosslinking by 6% indicates that the transpeptidation step of PG biosynthesis was inhibited by daptomycin in *E. faecalis*.

### Effects of daptomycin on the PG-stem and inter-bridge modifications

Each PG-repeat unit of *E. faecalis* contains four modifiable alanines: two d-Ala at the PG-stem, and two l-Ala of the inter-bridge structure (Fig. 5a). The terminal d-Ala-d-Ala in PG-stems can be sequentially cleaved by the d,d- and l,d-carboxypeptidases, resulting in modifications from pentapeptide- to tetrapeptide- and tripeptide-stem structures. In *E. faecalis*, approximately 85% of the total PG-repeat units are found with l-Ala-l-Ala inter-bridge structure^35^. To characterize the effects of daptomycin on the PG-stem and inter-bridge structures, the observed muropeptide ions were categorized based on their alanylation states, which refers to the total number of modifiable alanines within a muropeptide which include two d-Ala on the uncrosslinked acyl acceptor stem and two l-Ala on the acyl donor stem of a muropeptide^36^. Schematic representations of oligomers with different alanylation states are depicted in Fig. 5d. For example, a PG dimer with an alanylation state of + 2 corresponds to a 4–3 crosslinked dimer with intact bridge structure on both subunits and a pentapeptide with two d-Ala on an acceptor stem. Dimer with an alanylation state of + 1 will have an intact bridge structure and an uncrosslinked stem terminating with a tetrapeptide with one modifiable d-Ala. Alanylation state of 0 and -1 each have two possible structures for dimers: the first structure has a tripeptide acceptor stem with intact l-Ala-l-Ala bridge on both units, and the second possible structure has a pentapeptide stem on the acceptor stem but is missing l-Ala-l-Ala bridge on the donor stem. Alanylation state of -1 also corresponds to two possible dimer structures: 1) a dimer with a missing bridge on the donor stem and a tetrapeptide on an acceptor stem, or 2) a dimer with a tripeptide with one missing l-Ala bridge. Lastly, alanylation state of -2 is assigned to a dimer with a missing bridge unit on the donor stem and a tripeptide on an acceptor stem.

**Figure 7:**
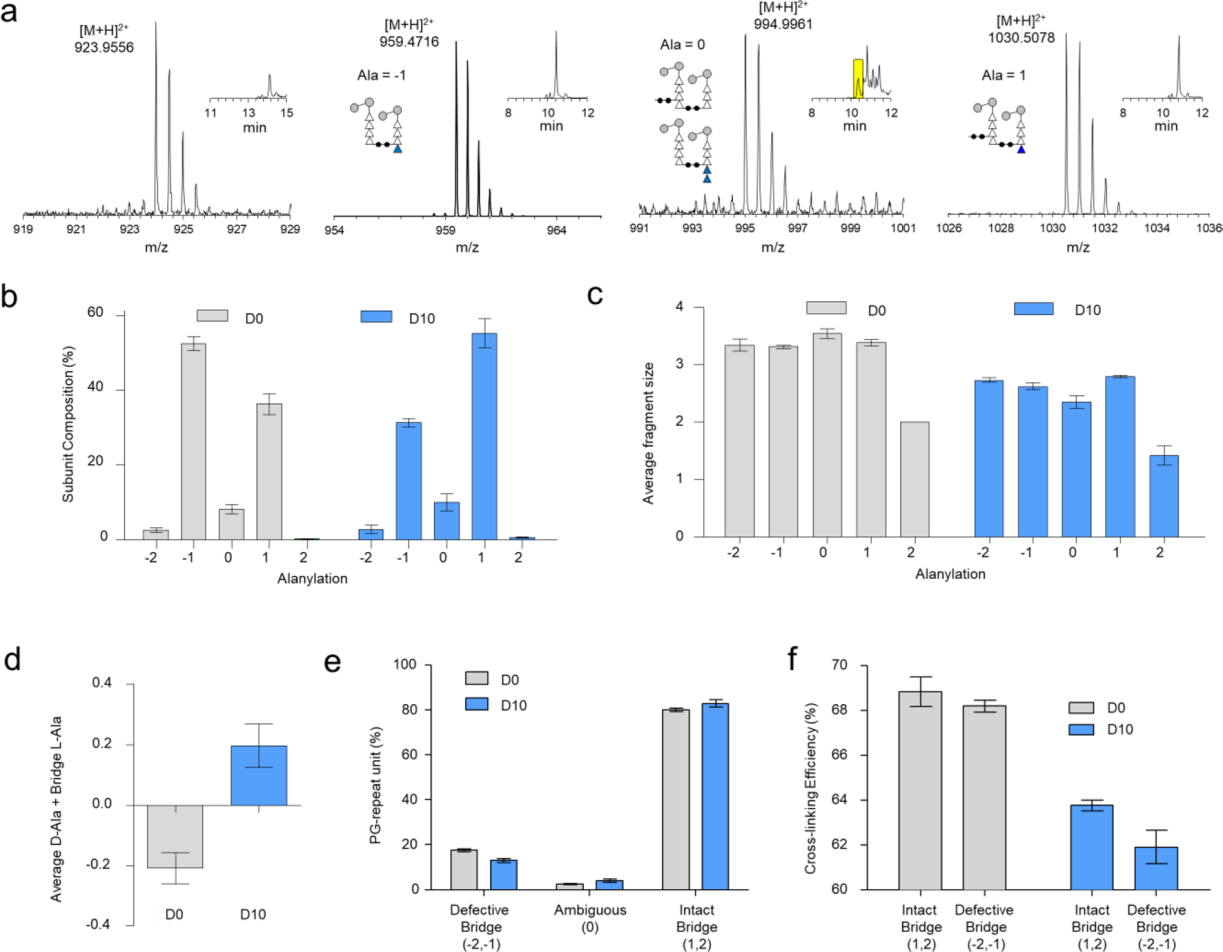
PG stem length and crosslinking bridge. (**a**) Mass spectra and corresponding extracted ion chromatograms (XIC) of muropeptide ions (dimers) with various peptide stem modifications from mutanolysin-digested isolated cell walls of untreated *E. faecalis*. The yellow box in the third-panel figure inset indicates the region of XIC selected for the displayed mass spectrum. (**b**) PG compositions categorized based on the alanylation state of muropeptides from cell walls of *E. faecalis* grown in the absence (D0, gray) and presence of daptomycin at 10 µg/mL (D10, blue). The percent PG-repeat unit composition based on alanylation is provided in Table S2a. (**c**) Distribution of average muropeptide fragment sizes based on their alanylation state. Addition of daptomycin significantly decreased the average muropeptide fragment size (Table S2b). (**d**) Proportion of alanylation state per subunit shows a higher abundance of PG with intact crosslinking bridges upon daptomycin addition, increasing from − 0.21 ± 0.05% under normal conditions to 0.20 ± 0.07% in the presence of daptomycin (n = 3) (Table S2c). (**e**) Relative abundances of PG units by bridge link. “Defective Bridge” represents the sum of XIC intensities of muropeptides with alanylation states of − 2 and − 1 (Fig. 7**b**), while “Intact Bridge” is the sum of muropeptides with alanylation states of + 1 and + 2 (Fig. 7**b**) (Table S2d). (**f**) Calculated PG crosslinking efficiencies of muropeptides with and without intact PG-bridge structure in *E. faecalis* cell walls grown in the absence (gray) and presence of daptomycin (blue bar). All error bars represent a 95% confidence interval (n = 3) (Table S2e).

Characterization of PG dimers with different alanylation states (-2, -1, 0, and + 1) in mutanolysin-digested isolated cell walls of *E. faecalis* grown without daptomycin is depicted in Fig. 7a. The figure is accompanied by the corresponding XIC shown as a figure inset. The integrated ion intensities of identified muropeptides are summed based on their alanylation states, and their normalized distributions are shown in Fig. 7b. In absence of daptomycin, approximately 89% of observed muropeptides have an alanylation state of either + 1 or -1. The most abundant muropeptides have an alanylation state of -1, accounting for 53% of the total, followed by the muropeptides with an alanylation state of + 1, constituting 36%. These findings suggest a high d,d-carboxypeptidase activity in *E. faecalis* as more than one-third of muropeptide fragments have alanylation states of + 1, characterized by a tetrapeptide in the uncrosslinked PG-stem and by the negligible presence of muropeptides with a pentapeptide stem structure (Ala = 2). In contrast, the activity of l,d-carboxypeptidase in *E. faecalis* appears to be low, as indicated by the low abundance of muropeptides with alanylation states of 0 and -2. With the addition of daptomycin, the abundance of muropeptides with an alanylation state of -1 decreased by approximately 5% to 31%. This reduction was offset by an increase in the abundance of muropeptides with an alanylation state of + 1 measured at 55.25% ± 3.91% (Fig. 7b). The presence of daptomycin also led to the reduction in the average muropeptide fragment size across all alanylation states (Fig. 7c), consistent with reduced PG crosslinking observed in Fig. 6b. Meanwhile, the abundance of muropeptides with an intact bridge structure showed an increase (Fig. 7d). These observations indicate that daptomycin inhibited the activities of transpeptidase and l,d-carboxypeptidase in *E. faecalis*.

The alanylation state also provides insight into the effect of daptomycin on the PG bridge link. Muropeptides with alanylation states of -2 and -1 correspond to PG with one or both l-Ala missing from its bridge, hence these muropeptides have a defective bridge structure. In contrast, muropeptides with alanylation state + 1 and + 2 have an intact l-Ala-l-Ala bridge on their acyl-doner stem. As shown in Fig. 7e, the addition of daptomycin did not significantly change the distribution of muropeptides based on their alanylation states. Approximately 80% of PG-repeat units in *E. faecalis* have an intact bridge attached (Fig. 7e) and this remains unchanged with the addition of daptomycin. Therefore, daptomycin did not interfere with the inter-bridge attachment for the maturation of lipid II. However, the PG of daptomycin-treated *E. faecalis* exhibited a decrease in ρ_CL_ for both muropeptides with and without a bridge structure (Fig. 7f). This is consistent with an inhibitory effect of daptomycin on transpeptidase activity.

### Effects of daptomycin on PG acetylation

**Figure 8:**
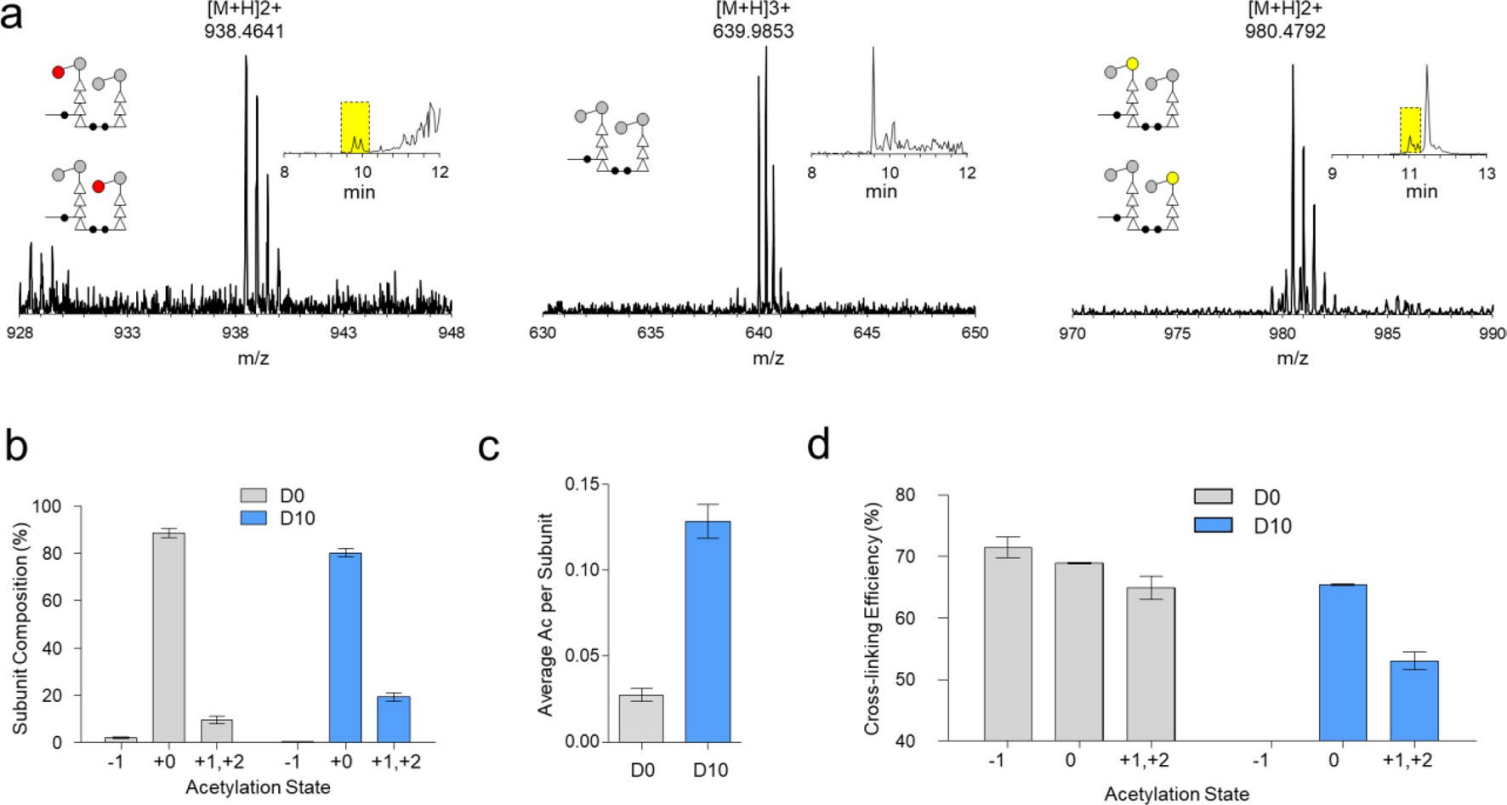
Effects of daptomycin on PG acetylation. (**a**) Representative mass spectra of PG dimers with a tripeptide stem from mutanolysin-digested isolated cell walls of *E. faecalis* with various acetylation states. Mass spectrum and schematic representation of PG dimers with an acetylation state of -1 are shown on the left, 0 in the middle, and + 1 on the right. Schematic representations of proposed muropeptide structures for each acetylation state and XIC are shown as insets. The yellow box in the panel inset indicates the region of XIC selected for the displayed mass spectrum. (**b**) Distributions of PG fragments by their acetylation states. Unmodified PG is predominant in the cell walls of both untreated (D0, gray) and daptomycin-treated *E. faecalis* (D10, blue) (Table S3a). An increase in the abundance of muropeptides with acetylation states of + 1 and + 2 is observed for the cell walls of daptomycin-treated *E. faecalis*. (**c**) Average acetylation per PG subunit. PG acetylation is increased in *E. faecalis* when treated with daptomycin (Table S3b). (**d**) Acetylation number and crosslinking efficiency. All error bars represent a 95% confidence interval (n = 3) (Table S3c).

We define the following PG acetylation states: -1 to represent the deacetylation, 0 for the unmodified PG fragment, and + 1 for the acetylation of the muropeptide disaccharide (Fig. 5e). The acetylation state was determined by observing a gain of 42.01 amu or a loss of 41.00 amu from the unmodified PG, corresponding to acetylation and deacetylation, respectively. Representative mass spectra of PG dimers with various acetylation states from the cell walls of untreated *E. faecalis* (D0) are shown in Fig. 8a, and the subunit composition analysis based on the acetylation states for muropeptides of D0 and D10 is shown in Fig. 8b. Following the daptomycin treatment, a large increase in PG acetylation by approximately 10% was observed, with the acetylation increasing from 9% in D0 to 19% in D10. This increase was accompanied by a sharp decline in the *N*-deacetylation of muropeptide in daptomycin-treated *E. faecalis* (Fig. 8b). Overall, the calculated average amount of acetylation per PG subunit in the cell walls of *E. faecalis* shows a sharp increase following the daptomycin treatment (Fig. 8c).

The increased PG acetylation following the daptomycin treatment preferentially occurred in PG with reduced crosslinking. To establish this correlation between the PG acetylation and crosslinking, the ρ_CL_ specific to each acetylation state of muropeptide species was calculated (Fig. 8d). Since the cell walls of daptomycin-treated *E. faecalis* were devoid of *N*-deacetylated muropeptides, ρ_CL_ for the acetylation state of -1 could not be determined. For muropeptides with acetylation states of + 1 and + 2, daptomycin-treated samples showed decrease in ρ_CL_ from 65% (D0) to 53% (D10). This 12% decrease in ρ_CL_ for acetylated muropeptides in daptomycin-treated *E. faecalis* is twice the average ρ_CL_ reduction calculated for all muropeptides, which was 6%. The high occurrence of PG acetylation on the muropeptides with low PG crosslinking suggests that acetylation could serve as a response mechanism to mitigate cell wall stress induced by daptomycin. As daptomycin reduces PG crosslinking, the newly synthesized PG in immature cell walls may be protected from autolysins through *O*-acetylation.

### Effects of daptomycin on 1,6-anhydrous ring formation at MurNAc

**Figure 9:**
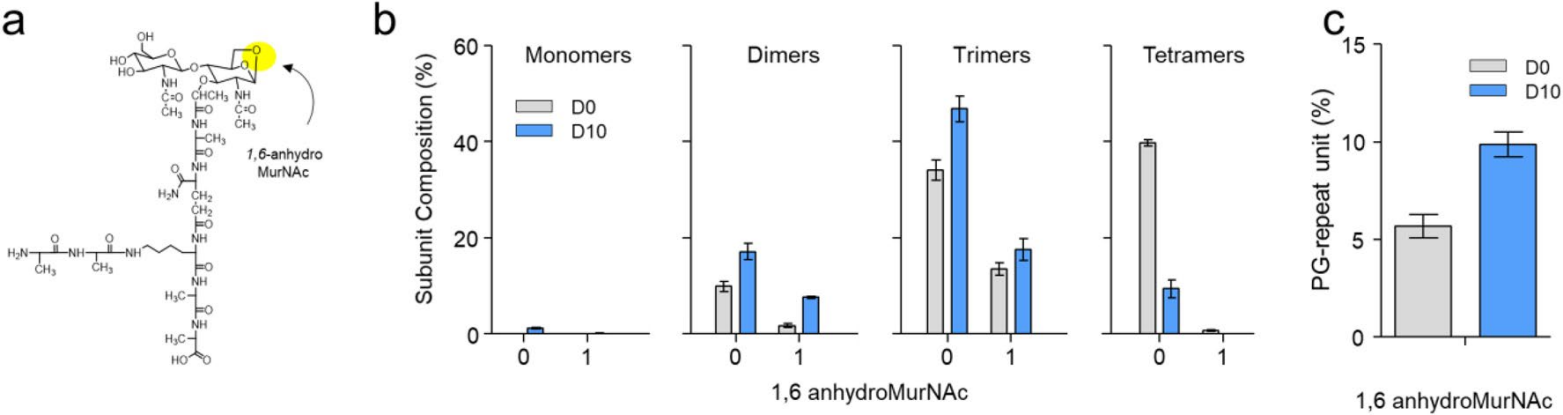
Effects of daptomycin on the composition of PG with a 1,6-anhydrous ring structure at MurNAc. (**a**) Chemical structure of a PG repeat unit with a 1,6-anhydrous ring at MurNAc (yellow circle). (**b**) Distribution of muropeptides containing a 1,6-anhydrous ring at MurNAc by the degree of oligomerization for *E. faecalis* grown in the absence (D0, gray bars) and presence of daptomycin at 10 µg/mL (D10, blue bars). The x-axis indicates the number of 1,6-anhydrous rings at MurNAc. All error bars represent a 95% confidence interval (n = 3). (**c**) The overall percentage of PG-repeat units with a 1,6-anhydrous ring at MurNAc. Following the addition of daptomycin, the percentage of muropeptides with a 1,6-anhydrous ring at MurNAc increased from 5.67 ± 0.60% to 9.88 ± 0.63%.

Cell walls undergo continuous remodeling during exponential growth with the help of lytic transglycosylases (LTs) to accommodate cell expansion and division^37^. LTs carry out this crucial role by cleaving the glycosidic bond between MurNAc and GlcNAc of the glycan chain. As a byproduct of this cleavage reaction, a 1,6-anhydrous ring structure is formed at the MurNAc (Fig. 9a)^38^. We hypothesize that in the absence of newly synthesized PG, sacculus remodeling by autolysins can lead to cell wall thinning (Fig. 3) and eventual cell death^29^. To determine whether daptomycin-induced cell wall thinning is caused in part by LTs, the change in PG composition of muropeptides containing a MurNAc 1,6-anhydro ring structure in *E. faecalis* following daptomycin treatment was analyzed. The muropeptides with a 1,6-anhydro ring structure were identified by a decrease in 20.03 amu from the unmodified PG. However, the specific location of the 1,6-anhydro ring structure within PG oligomers (dimers, trimer, and tetramers) was not determined. Figure 9b shows the muropeptide composition of monomer, dimer, trimer, and tetramers with and without a 1,6-anhydrous ring structure. Following daptomycin treatment, we observed an increase in dimers and trimers with a 1,6-anhydro ring structure at MurNAc. However, this increase was not observed in tetramers due to the substantial decrease in total tetramers caused by daptomycin inhibition of the transpeptidation step of PG biosynthesis, the total number of tetramers decreased dramatically (Fig. 6b). Consequently, the proportion of PG-repeat units with 1,6-anhydro ring structure in *E. faecalis* upon daptomycin treatment increased from 6 to 10% (Fig. 9c).

### PG O-acetylation and 1,6-anhydrous ring structure at MurNAc

LTs catalyze the glycan cleavage reaction by abstracting the proton from the C6-OH of MurNAc to generate C6 hydroxyl which nucleophilic attacks intramolecularly the C1 of MurNAc to form a 1,6-anhydrous ring structure^39^. This cleavage reaction is inhibited by the *O*-acetylation of C6-OH at MurNAc by preventing the generation of the nucleophile required for the catalysis. Therefore, PG O-acetylation interferes with the cell wall remodeling carried out by the LTs. In *Streptococcus pneumoniae*, the deletion of a gene responsible for PG O-acetylation has been shown to increase the activity of autolysin LytA^40^. Thus, we speculate that the increase in PG O-acetylation in daptomycin-treated *E. faecalis* (Fig. 8) may be a response to cell wall stress induced by daptomycin, to protect against further cell wall damage caused by autolysins. This response is consistent with our previous observation of a decrease in PG crosslinking accompanied by increased PG O-acetylation in vancomycin-resistant *E. faecalis* when grown in the presence of a sub-inhibitory concentration of vancomycin^33,41^.

### The mode of action of daptomycin and bacterial responses to daptomycin

Solid-state NMR analysis was performed on intact whole cells of *S. aureus* grown in a chemically defined media containing l-[ϵ-^15^N]Lys in the presence of a sub-inhibitory concentration of daptomycin. The ^15^N-NMR spectrum of daptomycin-treated *S. aureus* showed a decrease in the intensities of both lysyl-ε-amide resonance at 95 ppm and lysyl-ε-amine resonance at 10 ppm (Fig. 2c). The reduction in intensity at 95-ppm is consistent with cell wall thinning^26^ which has been observed for several antibiotics that inhibit cell wall biosynthesis including vancomycin^9,18,29,31,42,43^. Thus, the cell wall thinning in daptomycin-treated *S. aureus* is consistent with daptomycin inhibition of cell wall biosynthesis. However, the mode of action of daptomycin differs from that of vancomycin, as evident by the decreased intensity of the lysyl-ε-amine resonance at 10 ppm. This reduction indicates that Park’s Nucleotide does not accumulate in the cytoplasm of daptomycin-treated *S. aureus*. In contrast, bacteria treated with transglycosylase inhibitors that target lipid II accumulates in Park’s Nucleotide because lipid II sequestration prevents the recycling of bactoprenol phosphate necessary for the transport of PG precursors from the cytoplasmic to membrane^18,26,29,30,41,44^. Despite in vitro assays demonstrating of daptomycin binding to purified lipid II^17^,solid-state NMR finding shows that daptomycin in situ does not specifically target lipid II.

Our solid-state NMR result is consistent with earlier finding by Mengin-Lecreulx and coworkers, who used biochemical methods to determine that daptomycin-treated *Bacillus megaterium* showed inhibition of PG biosynthesis along with a depletion of cytoplasmic Park’s Nucleotide^45^. They attributed this effect to daptomycin-induced membrane pore formation, which resulted in the loss of electrochemical gradient potential that hindered the activity of amino acid transporters necessary for the biosynthesis of cytoplasmic PG precursors^46^. However, considering that membrane depolarization has been observed only at high daptomycin concentrations^9,11,15^, it remains uncertain whether the reduced Park’s Nucleotide in *S. aureus* treated with sub-inhibitory concentration of daptomycin is solely attributed to the loss of electrochemical gradient potential. Our TEM images of *S. aureus* at mid-exponential growth, after 90 min of growth at a sub-minimal inhibitory concentration of daptomycin, did not show any visible signs of aberrant cell morphology or membrane disruption (Fig. 2d). Nevertheless, solid-state NMR analysis of these cells provided evidence of PG biosynthesis inhibition that was accompanied by the depletion of Park’s Nucleotide.

**Figure 10:**
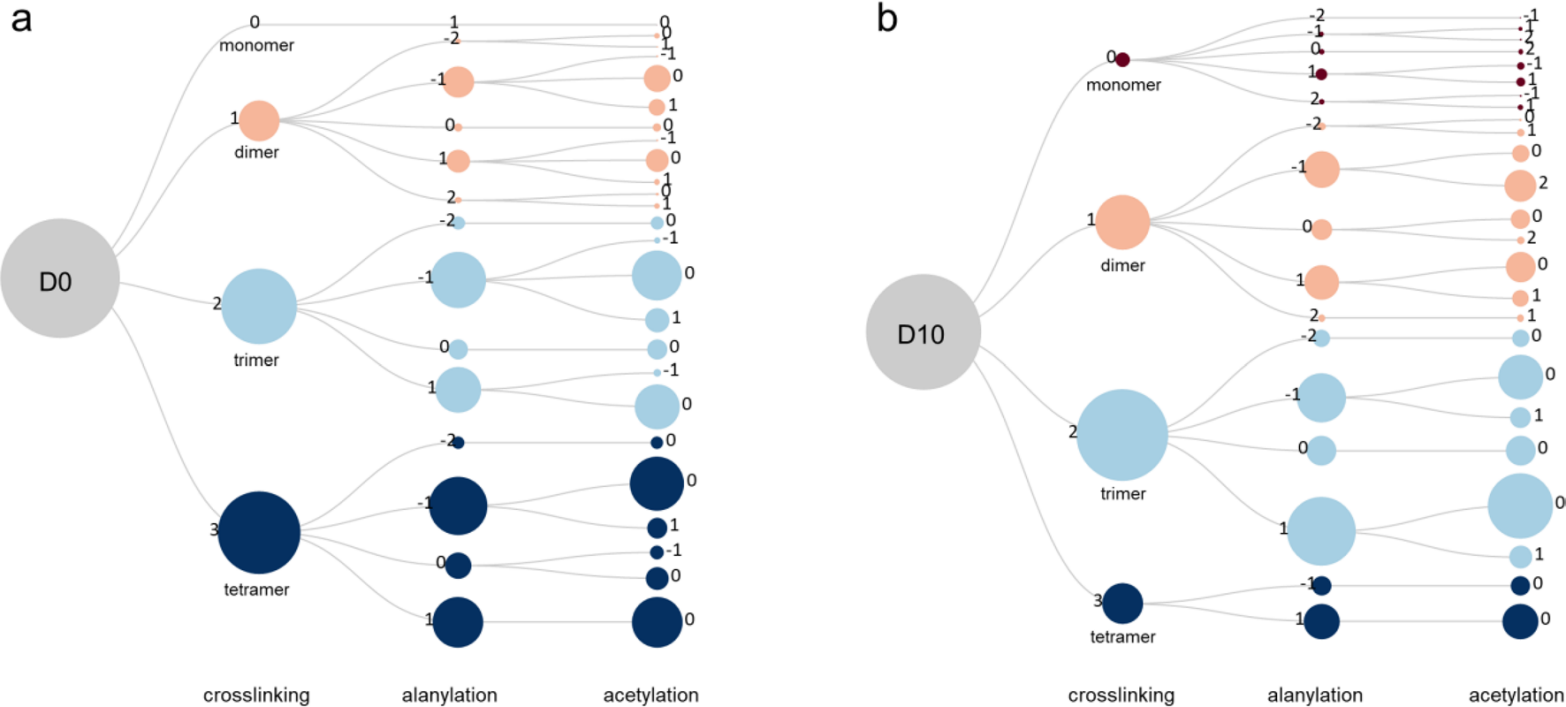
The peptidoglycan composition of *E. faecalis* undergoes a shift following the daptomycin treatment. To visualize this shift, linear dendrograms are used to represent the PG composition of (**a**) untreated and (**b**) daptomycin-treated *E. faecalis* (10 µg/mL) using quantified muropeptide ions shown in Fig. 5b. Each area within the circle represents the percentile muropeptide composition determined by integrating the XIC of the corresponding muropeptide ions. The first clustering of circles in the dendrogram is based on the crosslinking number (**c**), which is shown adjacent to the circles. To aid visualization, the circles belonging to each cluster are color-coded based on their crosslinking number. Monomers (c = 0) are presented by the color maroon, dimers (c = 1) by peach, trimers (c = 2) by aqua, and tetramers (c = 3) by indigo. The second clustering of circles is based on the alanylation states with values ranging from − 2 to + 2. The third clustering of circles is based on the acetylation states of each muropeptide. The circles are arranged in the dendrogram in descending order of values corresponding to the crosslinking number, alanylation state, and acetylation state.

To further investigate the impact of daptomycin on PG biosynthesis, we analyzed the mutanolysin-digested isolated cell walls of *E. faecalis* using LC–MS. The PG compositions of *E. faecalis* grown in the presence or absence of daptomycin are visualized as linear dendrograms using RAWGraph^47^ (Fig. 10). In these dendrograms, the size of each circle represents the muropeptide percentile composition, and the circles are clustered based on the observed muropeptide ions’ crosslinking number, alanylation state, and acetylation states (Fig. 5b). The circles are color-coded based on crosslinking numbers to aid visualization. A striking noticeable difference between the dendrograms illustrates the direct impact of daptomycin on PG biosynthesis. The most abundant muropeptides found in untreated cells are tetramers (Fig. 10a, indigo circles), but they nearly all disappear in daptomycin-treated cells. Concurrently, the daptomycin-treated cells show increases in monomers, dimers, and trimers (Fig. 10b), indicating a reduction in PG crosslinking. Overall, the cell walls of daptomycin-treated *E. faecalis* showed a substantial 6% reduction in PG crosslinking. Other notable changes in PG composition induced by daptomycin include increases in muropeptides with a 1,6-anhydrous ring structure and PG *O*-acetylation. Elevated levels of 1,6-anhydro ring structured muropeptides indicate that the cell walls of daptomycin-treated *E. faecalis* are highly susceptibility to LT degradation, possibly due to the changed PG structure and organization within the cell walls. The increase in PG *O*-acetylation may be a bacterial response to counter the deleterious effects of the autolysins, while the increase in muropeptides with a tetrapeptide stem structure indicates reduced l,d-carboxypeptidases activity.

Daptomycin exhibits a unique mode of action at sub-inhibitory concentrations by inhibiting PG biosynthesis. The evidence shows that daptomycin is not a transglycosylase inhibitor, ruling out lipid II binding, due to the absence of Park’s Nucleotide accumulation in daptomycin-treated bacteria. Furthermore, daptomycin interferes with the maturation of newly synthesized nascent PG, leading to cell walls with reduced PG crosslinking. This decoupling of transglycosylase from transpeptidase activities suggests a possible delocalization of either nascent PG or proteins involved in PG biosynthesis from the site of cell wall biosynthesis. When fluorescent-labeled daptomycin is introduced to *S. aureus*, it initially localizes to the divisional septum but eventually diffuses evenly throughout the cell surface^17^, indicating that daptomycin’s binding to its target, such as lipids in membrane or membrane rafts, may destabilizes its local environment. This destabilization can hinder the recruitment of essential enzymes required for cell wall biosynthesis. Although daptomycin does not inhibit the transglycosylation step of PG biosynthesis, it can disrupt PG crosslinking and maturation, potentially inhibit the biosynthesis of other biopolymers, such as teichoic acids, that are essential components of cell walls. Subsequently, daptomycin has been shown to inhibit the synthesis of both wall-teichoic and lipoteichoic acids^48^. Since teichoic acids have been shown to play a vital role in autolysins regulation^49–51^, the reduced presence of teichoic acids in daptomycin-treated bacteria may increase autolysin activity, which can contribute to the cell wall thinning. This is supported by the increased accumulation of 1,6-anhydro ring structured muropeptides in the cell walls of daptomycin-treated *E. faecalis*. Interestingly, the cell walls of daptomycin-treated *E. faecalis* show increased PG O-acetylation. While O-acetylation at the C6 position of MurNAc can prevent the LT cleavage of the glycan^52^; the C6 hydroxyl of MurNAc is also the site of attachment for anionic wall teichoic acids^52^. Thus, the increased synthesis of immature PG with reduced crosslinking, along with the inhibition of teichoic acid biosynthesis and increased LT activities, collectively compromise the structural integrity of the cell walls in daptomycin-treated bacteria.

## Materials and methods

### *S. aureus* growth condition

A starter culture of *S. aureus* (ATCC 6538P) grown overnight in 5 mL of trypticase soy broth at 37 °C was added (1% v/v) to two 1 L flasks, each containing 300 mL of *S. aureus* Standard Medium (SASM) supplemented with calcium chloride to a final concentration of 50 μg/mL^19^. Detailed protocol for the defined medium SASM has been described previously^26–28^. PG bridge links were labeled by adding [^13^C]Gly and l-[ϵ-^15^N]Lys to be incorporated into PG. Daptomycin was added at mid-exponential growth of *S. aureus* (OD_600_ 0.4) to final drug concentrations of 0, 20, or 40 μg/mL. Immediately following the addition of daptomycin, calcium chloride stock solution was added to SASM to a final concentration of 50 μg/mL to ensure the baseline activity of daptomycin. Cells were harvested after 90 min of incubation post daptomycin addition by centrifugation at 8,000 g for 10 min at 4 °C (Sorvall GS-3 rotor). The resulting pellets were washed twice with 50 mL of ice-cold 40 mM triethanolamine buffer, pH 7.0, resuspended in 10 mL of water, and then lyophilized.

### Solid-state NMR Spectrometer

The bridge linking in whole cells of *S. aureus* was measured by ^15^N CPMAS NMR performed on whole cells at 7.0 T (300 MHz for ^1^H, and 30 MHz for ^15^N) provided by 89-mm bore Oxford (Cambridge, U.K.) superconducting solenoids. Four-frequency transmission-line probe used in a 7.0-T spectrometer used a 14-mm long, 9-mm inner-diameter sample coil. The probe was equipped with Chemagnetics/Varian magic-angle spinning ceramic stator, and samples were spun at room temperature at 5 kHz (maintained within ± 2 Hz). Radio-frequency pulses were produced by 1-kW Kalmus, ENI, and American Microwave Technology power amplifiers, each under active control; π-pulse lengths were 10 µs for both ^13^C and ^15^N. Proton-nitrogen matched cross-polarization transfers were at 50 kHz for 2 ms. Proton dipolar decoupling during signal acquisition was 105 kHz. The re-cycle delay period was 2 s during which each amplifier produced a 300-μsec test pulse. The resulting diode-detected voltages were compared to the reference voltage previously calibrated for active control of the amplifiers^53^.

### Transmission Electron Microscopy

Overnight culture of *S. aureus* grown in BHI media was harvested at OD_600_ of 1.1 by centrifugation at 2,750 g for 20 min at 4 °C (Eppendorf Centrifuge 5810R). The pellet was resuspended in fresh BHI of twice the volume and daptomycin was added to a final concentration of 40 μg/mL After one-hour incubation at 37 °C, cells were pelleted and fixed by 0.5 mL of 2% paraformaldehyde / 2.5% glutaraldehyde (Polysciences Inc.) in 100 mM phosphate buffer, pH 7.2 for 3 h at room temperature. Samples were washed in phosphate-buffered saline and post-fixed in 1% osmium tetroxide (Polysciences Inc.) for 1 h. Samples were then rinsed extensively in distilled water before enbloc staining with 1% aqueous uranyl acetate (Ted Pella Inc.) for 1 h. Following additional several rinses in distilled water, samples were dehydrated in a graded series of ethanol and embedded in Eponate 12 resin (Ted Pella Inc.). Sections of 95 nm were cut with Leica Ultracut UCT ultramicrotome (Leica Microsystems Inc.), stained with uranyl acetate and lead citrate, and viewed on JEOL 1200 EX transmission electron microscope (JEOL USA Inc.).

### *E. faecalis* growth conditions

Starter culture of daptomycin-sensitive *E. faecalis* (OG1RF) grown in BHI broth at 37 °C with 180 RPM orbital shaking was used to inoculate flasks containing 90 mL of BHI broth (1% v/v) with 500 mg/L of CaCl_2_. Daptomycin (10 μg/mL) was added to the culture during the mid-exponential growth phase at OD_600_ of 0.6. Cells were harvested after 90 min of growth with the antibiotic by centrifugation at 4750 RPM for 10 min at 4 °C. Cell pellets were rinsed twice with deionized water, frozen, and then lyophilized.

### Membrane depolarization assay

The ATP-leakage assay was performed on an overnight culture of *E. faecalis* grown in BHI harvested at OD_600nm_ 1.5. The whole cells were pelleted and then resuspended in phosphate-buffered saline (PBS) supplemented with 20 mM Ca^2+^. Daptomycin was added to the suspension for final drug concentrations of 0, 1, 2, 5, 10, 20, 50, or 100 μg/mL. Daptomycin-treated cells were incubated for 20 min at 37 °C and the supernatant containing ATP was collected following brief centrifugation. After 10 min equilibration at room temperature, the amount of leaked ATP was quantified by adding 100 μL of CellTiter-Glo 2.0 reagents containing luciferin and luciferase (Promega, Madison WI) to an equal volume of supernatant. Luciferase catalyzes ATP-dependent oxidative decarboxylation of luciferin and releases photons at 560 nm. The luminescence was measured using Fluoroskan Ascent FL Luminometer (Thermo Scientific) with an integration time of 200 ms. The amount of leaked ATP was quantified by integrating the luminescence intensity.

### *E. faecalis* cell wall isolation and digestion for LC–MS

The procedure for cell wall isolation has been described previously^26^. In brief, lyophilized *E. faecalis* pellets from 90 mL growths were resuspended in PBS and sterilized by immersing them in a boiling water bath for 30 min. Cells were lysed by bead beating (Disruptor Genie, Scientific Industries) with 0.5 mm diameter glass beads for 8 one-min cycles with 1 min of rest in between agitation. Beads and other contaminants were removed using Steriflip 20 μm nylon vacuum filter (EMD Millipore). Crude cell wall pellets were resuspended in 2 mL PBS, to which 8 mL of 2% sodium dodecyl sulfate (SDS) solution was added, then placed in a boiling water bath for 30 min. Boiled cell wall pellets had SDS removed by dividing the pellets into microcentrifuge tubes and washing them with five 1 mL deionized water through centrifugation.

Isolated crude cell walls were resuspended in 2 mL of 50 mM Tris pH 8.0 buffer. DNase (200 μg) was added to the cell wall suspension and incubated at 37 °C for 24 h at 80 rpm, then trypsin was added (200 μg) prior to an additional 24 h of incubation. Cell walls were washed once and resuspended in 1 mL of Tris buffer. To hydrolyze β-1,4 glycosidic bonds in PG to generate PG fragments for the LC–MS compositional analysis, 0.50 KU of mutanolysin (Sigma-Aldrich) was added to the cell wall suspension at room temperature and the sample was incubated for 24 h. An additional 0.50 KU of mutanolysin was added to the mixture after the initial digestion for further 24 h digestion. The digested sample was frozen and lyophilized (Labconco). Lyophilized mutanolysin-digested cell walls were dissolved in 1 mL of 0.375 M sodium borate buffer (pH 9.0) prepared with HPLC-grade water, and samples were reduced by the addition of 10 mg of sodium borohydride (Fisher Scientific) in 960 μL borate buffer at room temperature for 30 min. The reduction was quenched by the addition of 125 μL of 85% phosphoric acid. Reduced samples were frozen at − 80 °C, and lyophilized. Lyophilized samples were resuspended in 1 mL of sample preparation buffer (1% trifluoroacetic acid), centrifuge filtered, and cleaned up for LC–MS using 100 μL Pierce C18 tips (Thermo Scientific). C18 tip was prepared by aspirating with a 100 µL of wetting buffer (1:1 acetonitrile (ACN): HPLC graded water v/v) followed by a 100µL of equilibrium buffer (0.1% Trifluoroacetic acid (TFA) in HPLC-grade water). The analytes were loaded to the C18 tip by slowly aspirating and dispensing 100 µL of prepared PG sample for 10 cycles. The tip was rinsed with 100 µL of the rinse buffer (0.1% TFA in 5% ACN:HPLC-grade water). Lastly, 100 µL of elution buffer was aspirated and the purified sample was dispensed into the sample vial. All LC–MS measurements were carried out in triplicate using mutanolysin-digested isolated cell walls prepared from a single 500 mL growth culture.

### Ultra-performance liquid chromatography

Mutanolysin-digested muropeptide fragments were chromatographically separated using NanoACQUITY Ultra Performance Liquid Chromatography System (Waters) on reverse-phase BEH C18 column (length: 100 mm, diameter: 75 μm) with bead size of 1.7 μm and pore size of 130 Å. Chromatographic separation of mutanolysin-digested PG was carried out by injecting 2 μL of the sample from a 5 μL sample loop to the column under the isocratic condition of 98% mobile phase A (0.1% formic acid) and 2% mobile phase B (90:10 acetonitrile: HPLC water (% v/v) with 0.1% formic acid added) for 5 min, then linear gradient to 50% mobile phase B was applied over 30 min for separation. The column was regenerated under an isocratic condition with 85% buffer B for 5 min, linear gradient to 98% buffer A for 1 min, then isocratic at 98% buffer A for 23 min. The flow rate throughout the separation was kept constant (0.6 μL/min).

### Mass spectrometry and LC–MS data analysis

Eluents were analyzed by Waters Synapt G2 High Definition Mass Spectrometer (HDMS)-Time-of-Flight (TOF) mass analyzer operating under positive ion mode. The sample was ionized by nanoflow electrospray ionization (ESI) with a spray voltage of 35 V and capillary voltage of 3.5 kV. The mass analyzer was optimized for the m/z range of 100–2000. Fibrinopeptide B (Glu-Fib) was used as an internal standard to correct the drift of the instrument. Raw mass spectrometry data analysis was read using MassLynx (Waters) and analyzed using an in-house MATLAB script (MathWorks).

## Supplementary Information

Supplementary Tables.

## Data Availability

All data generated during this study are included in this published article and its supplementary information files.
