# Supplementary material for: The effects of daptomycin on cell wall biosynthesis in Enterococcal faecalis
Source: Sci Rep. 2023 Jul 28;13:12227. doi: 10.1038/s41598-023-39486-8 (PMC10382475; doi:10.1038/s41598-023-39486-8)
Supplement: Supplementary file 1 — Supplementary Tables. [file 41598_2023_39486_MOESM1_ESM.docx]

**SUPPORTING INFORMATION**

**The Effects of Daptomycin on Cell Wall Biosynthesis in *Enterococcal faecalis***

*Binayak Rimal^1*^, James Chang^2^, Chengyin Liu^3^, Raiyan Rashid^3^, Manmilan Singh^4^, and Sung Joon Kim^3^*

^1^Institute of Biomedical Studies, Baylor University, Waco, TX 76706, USA.

^2^Baylor University, Department of Chemistry One Bear Place #97046, 76798, Waco, TX, USA.

^3^Howard University, Department of Chemistry, Washington, D.C. 20059, USA.

^4^Washington University, Department of Chemistry, St. Louis, MO 63110, USA.

*Current address: Division of Infectious Diseases, Johns Hopkins University School of Medicine, Baltimore, MD 21287, USA

Correspondence should be addressed: [sung.kim@howard.edu](mailto:sung.kim@howard.edu).

Running title: Daptomycin mode of action by solid-state NMR and LC-MS

Keywords: *daptomycin, E. faecalis, S. aureus, peptidoglycan, solid-state NMR, LC-MS, cell wall*

**Table S1.** **Observed fractions of peptidoglycan oligomers found in the cell walls of *E. faecalis* (OG1RF).**

**a.** Classification of mutanolysin-digested PG of *E. faecalis* based on the number of repeated disaccharide units.

| D0 | Average (%)^a^ | Standard deviation | CI^b^ |
| --- | --- | --- | --- |
| Monomers | 0.003 | 0.000 | 0.001 |
| Dimers | 11.869 | 0.457 | 1.134 |
| Trimers | 40.634 | 0.808 | 2.009 |
| Tetramers | 47.494 | 0.614 | 1.524 |

***^a^***An average determined from triplicate measurements and shown in percentile PG-repeat unit. ***^b^*** 95% confidence interval.

| D10 | Average (%)^a^ | Standard deviation | CI^b^ |
| --- | --- | --- | --- |
| Monomers | 1.697 | 0.084 | 0.208 |
| Dimers | 22.993 | 0.695 | 1.726 |
| Trimers | 62.706 | 1.609 | 3.997 |
| Tetramers | 12.604 | 0.996 | 2.474 |

***^a^***An average determined from triplicate measurements and shown in percentile PG-repeat unit. ***^b^*** 95% confidence interval.

**b.** Calculated average muropeptide size for daptomycin untreated (D0) and treated (D10) *E. faecalis*

|  | Average^a^ | Standard deviation | CI^b^ |
| --- | --- | --- | --- |
| D0 | 3.356 | 0.007 | 0.018 |
| D10 | 2.685 | 0.009 | 0.021 |

***^a^***An average determined from triplicate measurements and shown in percentile PG-repeat unit. ***^b^*** 95% confidence interval.

**c.** Calculated PG cross-linking efficiency for *E. faecalis*

|  | Average (%)^a^ | Standard deviation | CI^b^ |
| --- | --- | --- | --- |
| D0 | 68.644 | 0.086 | 0.214 |
| D10 | 62.753 | 0.119 | 0.296 |

***^a^***An average determined from triplicate measurements and shown in percentile PG-repeat unit. ***^b^*** 95% confidence interval.

**Table S2.** **Observed fractions of peptidoglycan alanylation found in the cell walls of *E. faecalis*.**

**a.** Proportion of PG by the presence of L-Ala Bridge and terminal peptide stem lengths under normal condition and with daptomycin.

| D0 | Average (%)^a^ | Standard deviation | CI^b^ |
| --- | --- | --- | --- |
| -2 | 2.605 | 0.255 | 0.634 |
| -1 | 52.549 | 0.727 | 1.806 |
| +0 | 8.216 | 0.473 | 1.176 |
| +1 | 36.339 | 1.139 | 2.831 |
| +2 | 0.292 | 0.042 | 0.105 |

***^a^***An average determined from triplicate measurements and shown in percentile PG-repeat unit. ***^b^*** 95% confidence interval.

| D10 | Average (%)^a^ | Standard deviation | CI^b^ |
| --- | --- | --- | --- |
| -2 | 2.757 | 0.454 | 1.129 |
| -1 | 31.296 | 0.450 | 1.118 |
| +0 | 10.069 | 0.904 | 2.246 |
| +1 | 55.248 | 1.575 | 3.913 |
| +2 | 0.629 | 0.075 | 0.185 |

***^a^***An average determined from triplicate measurements and shown in percentile PG-repeat unit. ***^b^*** 95% confidence interval.

**b.** Addition of daptomycin markedly decreased the average fragment size.

| D0 | Average^a^ | Standard deviation | CI^b^ |
| --- | --- | --- | --- |
| -2 | 3.346 | 0.043 | 0.106 |
| -1 | 3.314 | 0.012 | 0.029 |
| 0 | 3.544 | 0.033 | 0.082 |
| 1 | 3.386 | 0.022 | 0.055 |
| 2 | 2.000 | 0.000 | 0.000 |

***^a^***An average determined from triplicate measurements and shown in percentile PG-repeat unit. ***^b^*** 95% confidence interval.

| D10 | Average^a^ | Standard deviation | CI^b^ |
| --- | --- | --- | --- |
| -2 | 2.730 | 0.015 | 0.037 |
| -1 | 2.617 | 0.024 | 0.060 |
| 0 | 2.283 | 0.045 | 0.113 |
| 1 | 2.790 | 0.009 | 0.021 |
| 2 | 1.417 | 0.067 | 0.166 |

***^a^***An average determined from triplicate measurements and shown in percentile PG-repeat unit. ***^b^*** 95% confidence interval.

**c.** Proportion of average alanine per subunit

|  | Average^a^ | Standard deviation | CI^b^ |
| --- | --- | --- | --- |
| D0 | -0.208 | 0.021 | 0.051 |
| D10 | 0.197 | 0.029 | 0.073 |

***^a^***An average determined from triplicate measurements and shown in percentile PG-repeat unit. ***^b^*** 95% confidence interval.

**d.** Relative proportion of defective bridge per PG-repeat unit. Defective bridge includes the fragments with either one or both L-ALA bridge missing.

| D0 | Average (%)^a^ | Standard deviation | CI^b^ |
| --- | --- | --- | --- |
| Defective bridge (-2,-1) | 17.541 | 0.227 | 0.564 |
| Ambiguous | 2.405 | 0.116 | 0.287 |
| Intact Bridge (1,2) | 80.054 | 0.286 | 0.711 |

***^a^***An average determined from triplicate measurements and shown in percentile PG-repeat unit. ***^b^*** 95% confidence interval.

| D10 | Average (%)^a^ | Standard deviation | CI^b^ |
| --- | --- | --- | --- |
| Defective bridge (-2,-1) | 12.971 | 0.405 | 1.006 |
| Ambiguous | 4.030 | 0.315 | 0.782 |
| Intact Bridge (1,2) | 82.999 | 0.693 | 1.722 |

***^a^***An average determined from triplicate measurements and shown in percentile PG-repeat unit. ***^b^*** 95% confidence interval.

**e.** Calculated PG cross-linking efficiency of intact versus defective bridge for *E. faecalis*

| D0 | Average (%)^a^ | Standard deviation | CI^b^ |
| --- | --- | --- | --- |
| Intact Bridge (1,2) | 68.848 | 0.265 | 0.659 |
| Defective bridge (-2,-1) | 68.195 | 0.110 | 0.273 |

***^a^***An average determined from triplicate measurements and shown in percentile PG-repeat unit. ***^b^*** 95% confidence interval.

| D10 | Average (%)^a^ | Standard deviation | CI^b^ |
| --- | --- | --- | --- |
| Intact Bridge (1,2) | 63.767 | 0.095 | 0.235 |
| Defective bridge (-2,-1) | 61.914 | 0.299 | 0.742 |

***^a^***An average determined from triplicate measurements and shown in percentile PG-repeat unit. ***^b^*** 95% confidence interval.

**Table S3.** **Observed fractions of peptidoglycan acetylation found in the cell walls of *E. faecalis*.**

**a.** Proportion of PG fragments by acetylation state for untreated and daptomycin-treated peptidoglycan

| D0 | Average (%)^a^ | Standard deviation | CI^b^ |
| --- | --- | --- | --- |
| -1 | 2.033 | 0.125 | 0.310 |
| +0 | 88.485 | 0.796 | 1.978 |
| +1,+2 | 9.482 | 0.696 | 1.728 |

***^a^***An average determined from triplicate measurements and shown in percentile PG-repeat unit. ***^b^*** 95% confidence interval.

| D10 | Average (%)^a^ | Standard deviation | CI^b^ |
| --- | --- | --- | --- |
| -1 | 0.498 | 0.028 | 0.070 |
| +0 | 80.162 | 0.701 | 1.743 |
| +1,+2 | 19.340 | 1.108 | 2.751 |

***^a^***An average determined from triplicate measurements and shown in percentile PG-repeat unit. ***^b^*** 95% confidence interval.

**b.** Proportion of average acetylation per subunit shows that *E. faecalis* in response to daptomycin increases in acetylation

|  | Average (%)^a^ | Standard deviation | CI^b^ |
| --- | --- | --- | --- |
| D0 | 0.027 | 0.002 | 0.003 |
| D10 | 0.130 | 0.132 | 0.327 |

***^a^***An average determined from triplicate measurements and shown in percentile PG-repeat unit. ***^b^*** 95% confidence interval.

**c.** PG Acetylation and crosslinking efficiency

| D0 | Average (%)^a^ | Standard deviation | CI^b^ |
| --- | --- | --- | --- |
| -1 | 71.466 | 0.681 | 1.693 |
| 0 | 68.972 | 0.048 | 0.119 |
| +1,+2 | 64.945 | 0.750 | 1.862 |

***^a^***An average determined from triplicate measurements and shown in percentile PG-repeat unit. ***^b^*** 95% confidence interval.

| D10 | Average (%)^a^ | Standard deviation | CI^b^ |
| --- | --- | --- | --- |
| -1 | 0.000 | 0.000 | 0.000 |
| 0 | 65.482 | 0.056 | 0.140 |
| +1,+2 | 53.048 | 0.580 | 1.440 |

***^a^***An average determined from triplicate measurements and shown in percentile PG-repeat unit. ***^b^*** 95% confidence interval.
